# Mild thermotherapy and hyperbaric oxygen enhance sensitivity of TMZ/PSi nanoparticles via decreasing the stemness in glioma

**DOI:** 10.1186/s12951-019-0483-1

**Published:** 2019-04-01

**Authors:** Xiaofan Zeng, Qi Wang, Xuan Tan, Le Jia, Yuwei Li, Mingdi Hu, Zhijie Zhang, Xicheng Bai, Yanhong Zhu, Xiangliang Yang

**Affiliations:** National Engineering Research Center for Nanomedicine, College of Life Science and Technology, Huazhong University of Science and Technology, No. 1037 Luoyu Road, Wuhan, 430074 People’s Republic of China

**Keywords:** Glioma, Photothermal therapy, Hyperbaric oxygen, Porous silicon nanoparticles, Stemness

## Abstract

**Background:**

Glioma is a common brain tumor with a high mortality rate. A small population of cells expressing stem-like cell markers in glioma contributes to drug resistance and tumor recurrence.

**Methods:**

Porous silicon nanoparticles (PSi NPs) as photothermal therapy (PTT) agents loaded with TMZ (TMZ/PSi NPs), was combined with hyperbaric oxygen (HBO) therapy in vitro and in vivo. To further investigate underlying mechanism, we detected the expression of stem-like cell markers and hypoxia related molecules in vitro and in vivo after treatment of TMZ/PSi NPs in combination with PTT and HBO.

**Results:**

NCH-421K and C6 cells were more sensitive to the combination treatment. Moreover, the expression of stem-like cell markers and hypoxia related molecules were decreased after combination treatment. The in vivo results were in line with in vitro. The combination treatment presents significant antitumor effects in mice bearing C6 tumor compared with the treatment of TMZ, PTT or TMZ/PSi NPs only.

**Conclusion:**

These results suggested the TMZ/PSi NPs combined with HBO and PTT could be a potential therapeutic strategy for glioma.

**Electronic supplementary material:**

The online version of this article (10.1186/s12951-019-0483-1) contains supplementary material, which is available to authorized users.

## Background

Gliomas are the most common brain tumors with a high mortality rate found in humans in Europe and the US [1, 2]. Surgery followed by chemotherapy or radiotherapy is the standard therapy strategy for glioma [3]. However, patients still exhibit a poor prognosis, with a mean survival time lower than 15 months [4, 5]. Increasing evidences have indicated the existence of a small population of glioma cells with stem cell properties, referred to as glioma stem-like cells, which contribute to therapy resistance, poor prognosis, and tumor recurrence [6, 7].

Hypoxia is an important characteristic of solid tumors and plays a significant role in stem-like cell development [8]. Hypoxia can lead to breast cancer stem cell (CSC) expansion [9]. Hypoxia significantly favored ADMSC proliferation and preserved the expression of stemness genes, i.e. Nanog and SOX2 [10]. Hypoxia is also a distinct feature in glioma. In the absence of serum, hypoxia induced C6 cells to dedifferentiate to a CSCs phenotype [11]. Clinically used anti-tumor drug TMZ against glioma increases the medial survival of the patient for only several months, which may happen due to chemoresistance under the hypoxia related environment [12, 13]. HBO could overcome the hypoxia microenvironment in solid tumor and increase the sensitivity of tumor cell to chemotherapy [14, 15].

Thermotherapy has long been used as a treatment method for cancer, but it is difficult to treat patients without damaging healthy cells. Among different thermotherapies, mild thermotherapy (40–44 °C) can enhance the drug effects and is more acceptable by patients [16, 17]. Heating rodent tumors at 40–42 °C was found to increase the blood flow and partial pressure of oxygen in the tumors. The increased blood flow caused by mild heat may improve the delivery of chemotherapy drugs to tumor cells [18]. Combining photothermal therapy (PTT) with chemotherapy is an interesting research direction in nano-medicine [19]. Nanodrug-mediated thermotherapy can eliminate CSCs [20]. Porous silicon (PSi) can be utilized as a therapeutic agent that generates mild heat upon exposure to NIR light [21]. Thermotherapy based on PSi under NIR light irradiation in combination with chemotherapy is an efficient technique to reduce cancer cells resistance [22–24]. Here, we hypothesized that the mild thermotherapy caused by PSi combined with HBO could increase the oxygen supply in the tumors and enhance chemosensitivity in tumor stem cells.

**Scheme 1:**
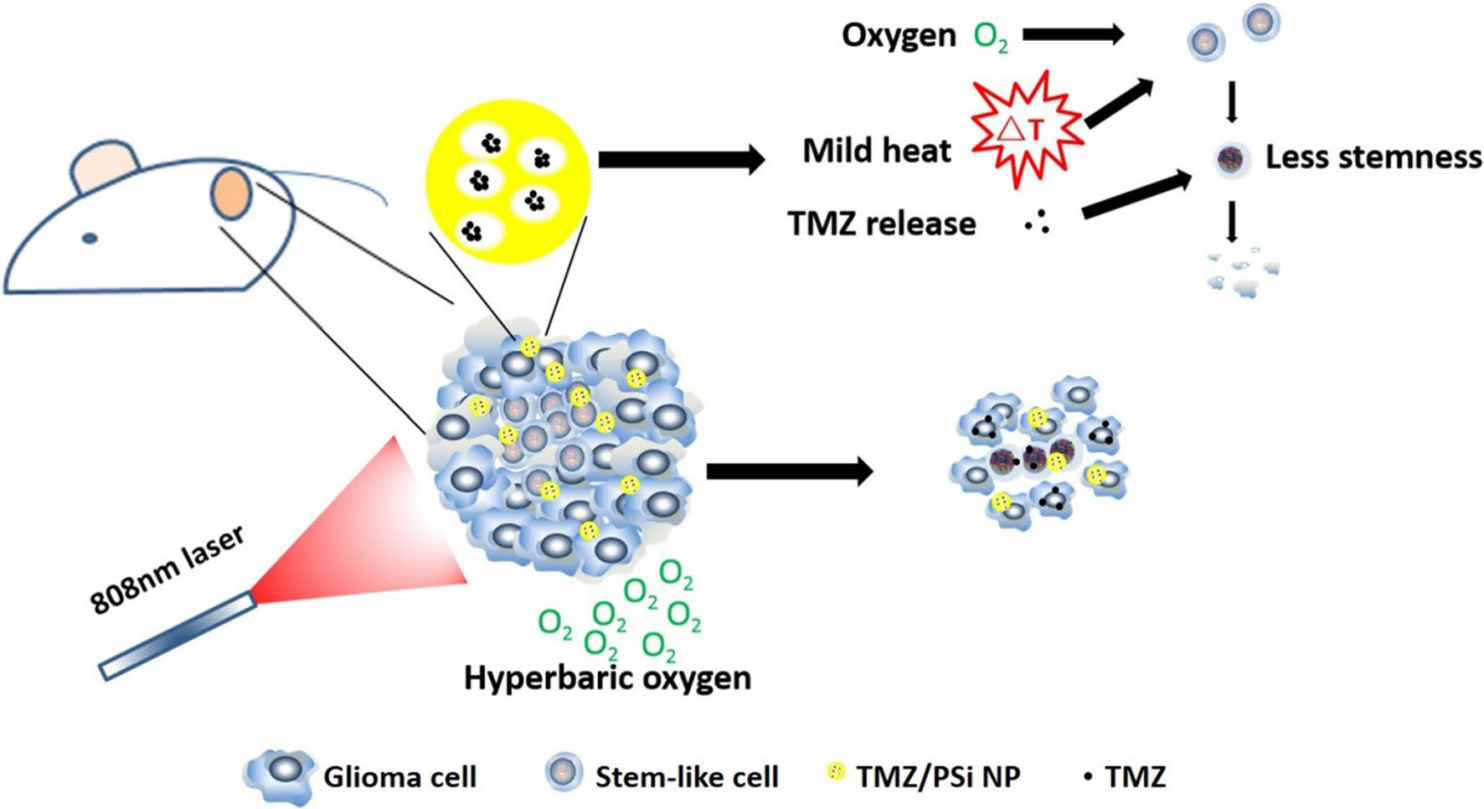
Scheme of TMZ/PSi nanomedicine combined with mild photothermal therapy and hyperbaric oxygen in glioma. The mice bearing tumor were administered intra-tumor injection with TMZ/PSi NPs followed with PTT and HBO treatments. The combination treatment reduced the stemness and enhanced the sensitivity of glioma tumor cells to TMZ

In this study, PSi loaded with TMZ for chemo-photothermal therapy, was further combined with HBO therapy to reduce self‑renewal of glioma stem-like cells and inhibit glioma growth (Scheme 1).

## Methods

### Materials

The boron-doped p-type silicon wafers were obtained from Virginia Semiconductor, Inc. (VA, USA) [15]. Drug TMZ was purchased from Aladdin Reagent Co. Ltd. (Shanghai, China). Other chemicals were of analytic grade. The experimental hyperbaric oxygen (HBO) animal chamber was purchased from Weifang Huaxin Oxygen Industry Co., Ltd (Weifang, China).

Rat glioma C6 cell line was preserved in our lab. Glioma stem cell line NCH-421K used in this study was kindly provided by the Neurosurgery Laboratory of Tongji Medical College, Huazhong University of Science and Technology. BALB/c-nude mice (male, 16–18 g) were purchased from Beijing Wei Tong Li Hua experimental animal Co., Ltd (Beijing, China). The animal protocol was approved by the Animal Experimentation Ethics Committee of College of Life Science and Technology, Huazhong University of Science and Technology.

### Preparation of PSi and TMZ/PSi NPs

Preparation of PSi NPs was performed as described previously [25]. TMZ/PSi NPs were prepared according our report with a little modification [15]. Briefly, TMZ dissolved in 5% phosphoric acid/methanol solution was added into the NPs under stirring overnight. TMZ-loaded PSi NPs (TMZ/PSi NPs) were obtained by ultrafiltration centrifugation and washed three times. Ultraviolet spectrophotometer was used to measure the amount of TMZ/PSi NPs.

### Characterization of PSi and TMZ/PSi NPs

The size and morphology were characterized by TEM (JEM-2010; JEOL, Tokyo, Japan). An 808 nm NIR laser (Changchun radium Photoelectric Technology Co., Ltd., China) was used to investigate the photothermal conversion capability of PSi and TMZ/PSi NPs.

### Drug release test

TMZ release at 37 °C or 42 °C was performed according to previous report [15, 26].

### HBO treatment

HBO therapy was performed at a pressure of 2.5 ATM according to our previous work [14].

### Photothermal therapy (PTT) treatment

Photothermal therapy (PTT) treatment was conducted with 808 nm NIR laser at 0.6 W/cm^2^ for 20 min. During irradiation, the temperature was monitored using thermography (E50, FLIR Systems Inc, USA). After treatment with TMZ/PSi, PTT was carried out for 20 min followed by HBO treatment in the combination treatment (PTT + HBO) groups.

### Cell culture

NCH-421K cells were cultured in DMEM/F12 medium (Hyclone) supplemented with 20% BIT (Stemcell Technologies), 10 ng/mL basic fibroblast growth factor (bFGF, Peprotech), 10 ng/mL epidermal growth factor (EGF, Peprotech). C6 cells were cultured with DMEM medium (Hyclone) supplemented with 10% FBS (Gibco).

### Cell viability assay of TMZ

Different dose of TMZ (50, 100, 200, 400, 800, 1600 μM) were used to test cell viability. NCH-421K or C6 Cells (8 × 10^3^ cells/well) were plated into 96-well plates and incubated at 37 °C with 5% CO_2_ overnight before adding TMZ. Cell viability was detected at 24, 48 and 72 h using the CCK-8 after different treatments respectively.

### Evaluation of TMZ/PSi on cell viability under different treatments

One critical toxicity dose of TMZ (400 μM) was used to assess the TMZ/PSi NPs effects on cancer stem-like cells. Cells were divided into PSi, TMZ or TMZ/PSi NPs (with or without PTT) groups at the different oxygen concentrations, respectively. NCH-421K cells and C6 cells were cultured in normoxia and then treated under 100% O_2_ for 90 min/day after adding drugs. After incubation for 72 h, SRB method was used to evaluate the cell viability [15].

### Assay of colony formation

NCH-421K cells at a density of 2.5 × 10^5^ cells/well were plated into 12-well plates and cultured overnight. Cells were treated with 100 μM TMZ or TMZ/PSi NPs for 24 h. Then the cells were harvested and countered, plated at a density of 2 × 10^4^ cells/well into 24-well plates for shape observation and the surviving fraction in 24-well plates was taken a picture on day 1, 4 and 7. 1 × 10^2^ cells/well was plated into 96-well plates for countering colony number and the number of tumorspheres in each well was recorded on day 7.

### mRNA and Western-blot analysis

For qRT-PCR assays, cell and tumor tissue RNA was extracted according the manual of PrimeScript RT reagent Kit (Takara Biotechnology Co., Ltd., China) and Primers were present in Additional file 1: Table S1. The StepOnePlus Real Time PCR System (Applied Biosystems, Foster City, CA, USA) was used. The resulting data were analyzed with the comparative cycle threshold (CT) value for relative gene expression quantification relative to GAPDH.

Cell proteins were extracted using normal method [7]. After blocking, antibody against HIF-1α (Abcam, UK), Nestin, SOX2, VEGF and GAPDH (Proteintech, China), were added, respectively.

### In vivo antitumor effects of combination treatment

To establish tumor bearing model, 3 × 10^6^ C6 cells were subcutaneously inoculated into a nude mouse. When tumor volume was above 75 mm^3^, the mice were randomly divided into six groups: the control group, PSi group, TMZ group, PTT group, TMZ/PSi group, and TMZ/PSi combined with PTT and HBO group respectively. According to our previous report, HBO alone has no effects on C6 glioma [15]. Therefore, HBO alone group was not included in this study. Then the mice bearing tumors were intratumor injected with TMZ or TMZ/PSi NPs (5 mg/kg TMZ) followed with PTT and HBO treatment on day 1, 4, 7, and 10 in the related groups, respectively. The sizes of the tumors were recorded every 2 days. The formula: tumor volume (mm^3^) = 0.5 × length (mm) × [width (mm)]^2^, was used to calculate the tumor volume. At the end of animal experiment, the mice were sacrificed, and tumor tissues and other organs were collected.

### Immunohistochemistry and mRNA analysis of tumor tissue

The immunohistochemistry analysis was performed. Rabbit polyclonal Nestin and SOX2 antibody (Servicebio, China) were used to stain stemness marker of the tumor tissue, respectively. Rabbit polyclonal HIF-1α, and VEGF antibody (Servicebio, China) were used to stain hypoxia marker and vascularization of the tumor tissue, respectively. Rabbit monoclonal Ki-67 antibody (Servicebio, China) was used for analyzing cell proliferation.

Tumor tissue RNA was extracted according the manual of PrimeScript RT reagent Kit (Takara Biotechnology Co., Ltd., China) for qRT-PCR assays.

### Statistical analysis

The data were expressed as mean ± SEM. Statistical significance in all experiment was determined using one-way ANOVA followed by a Student’s test for multiple comparison tests. Statistical analysis was analyzed using statistical software (IBM SPSS Statistics 20, USA).

## Results and discussion

### Characterization, photothermal effect and in vitro drug release

**Fig. 1:**
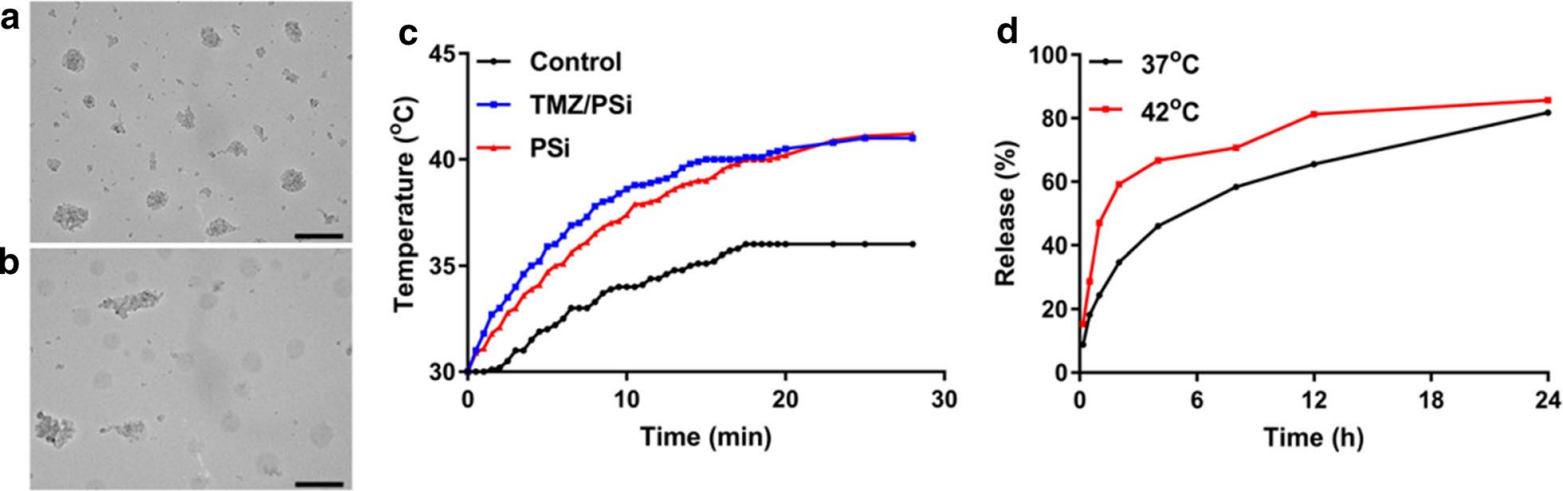
Characterization, photothermal effect and in vitro drug release. **a**, **b** TEM images of PSi and TMZ/PSi, bar: 200 nm. **c** Temperature increase curve of PSi and TMZ/PSi. **d** Drug release of TMZ/PSi at different temperatures

The irregular shapes of PSi and TMZ/PSi NPs were shown and the average size of PSi and TMZ/PSi NPs was about 90 nm and 140 nm under transmission electron microscope respectively (Fig. 1a, b). The DLS sizes of NPs remained stable for 1 week at room temperature (Additional file 1: Figure S1). The surface temperature of PSi and PSi/TMZ after exposure to 808 nm NIR laser at 0.6 W/cm^2^ was measured using a thermometer (Fig. 1c). The temperature reached to 42 °C approximately upon NIR laser irradiation and remained the temperature thereafter. TMZ release was about 80% within 24 h (Fig. 1d). An increasing release was observed at the beginning of 20 min after exposure to NIR laser irradiation, which illustrates that hyperthermia could accelerate drug release.

### Effects of TMZ and TMZ/PSi NPs in combination with PTT and HBO on stem-like cell viability

**Fig. 2:**
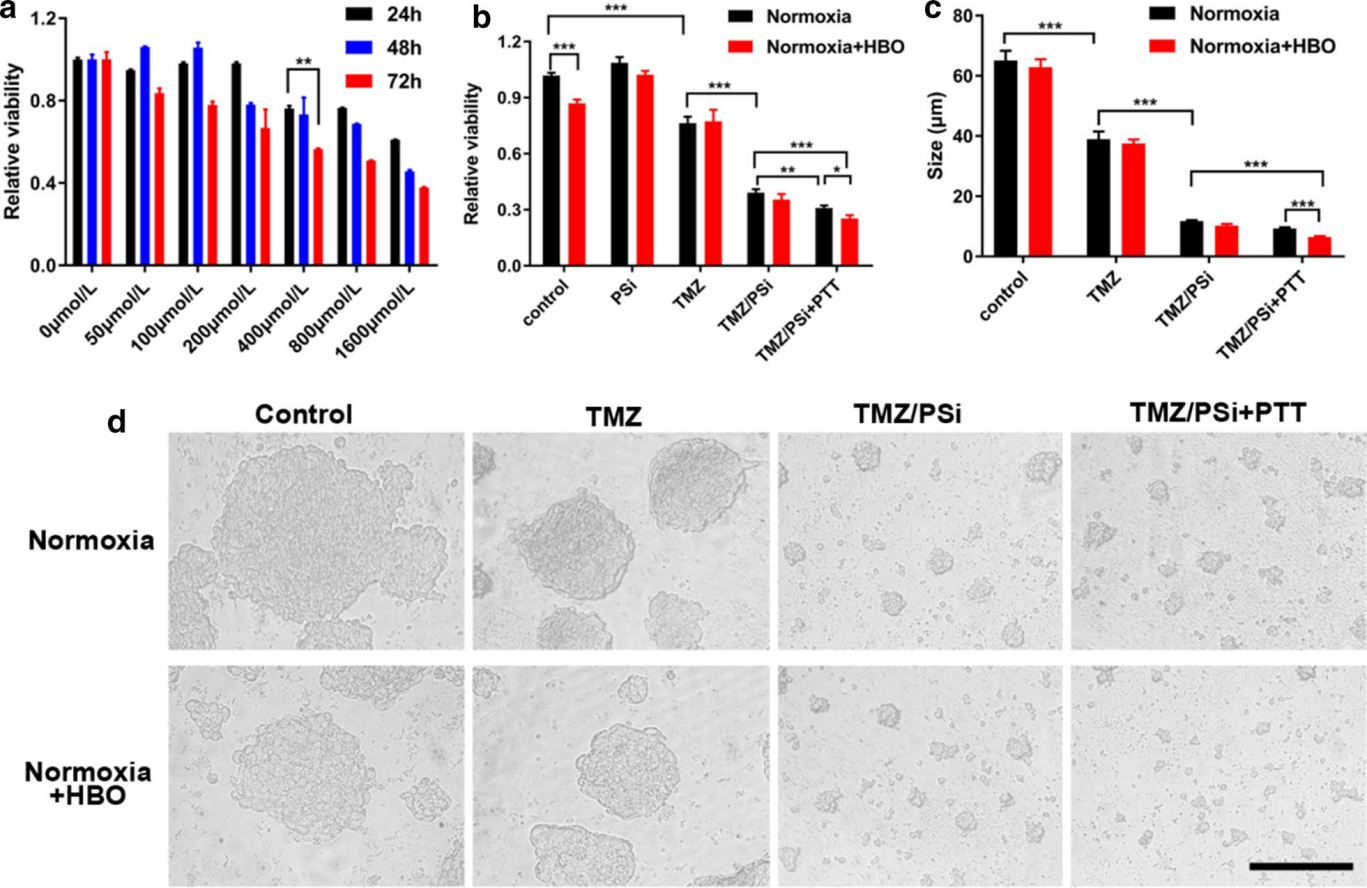
Effects of TMZ and TMZ/PSi NPs combined with PTT and HBO on glioma stem cell viability. **a** Viability of NCH-421K cells after treatments with different TMZ concentrations (n = 5; **P < 0.01). **b** Viability of NCH-421K cells after different treatments (n = 5; *P < 0.05, **P < 0.01, ***P < 0.001). **c**, **d** Tumorsphere size and shape after different treatments (n = 5; ***P < 0.001). NCH-421K cells were cultured in normoxia and then treated under 100% O_2_ for 90 min/day after adding drugs. After incubation for 72 h, CCK-8 or SRB method was used to evaluate the cell viability. Bar: 50 μm

The stem-like cell character (stemness) has been thought to be closely associated with tumor progression and therapeutic resistance [27]. Glioma stem cells (GSCs) are increasingly recognized as playing important roles in the chemoresistance of glioma [28]. To demonstrate tumor stem-like cell viability affected by TMZ/PSi or TMZ, glioma stem cell line NCH-421K (GSCs) were cultured in vitro under normoxia condition. As shown in Fig. 2a and Additional file 1: Figure S2, NCH-421K cell viability was significantly decreased when treated with TMZ at 400 μM.

Furthermore, the effect of TMZ/PSi NPs containing 400 μM of TMZ with or without HBO and PTT treatments on GSCs and C6 cells was studied (Additional file 1: Figure S3). The decrease of cell viability treated with TMZ/PSi NPs was observed after combination treatment with PTT and HBO compared to that without PTT or HBO treatment (Fig. 2b). Similarly, after treatment with same dose of TMZ/PSi, cell viability decreased significantly compared to free TMZ alone. Compared with TMZ/PSi, the cell viability was decreased both in TMZ/PSi + HBO group (P = 0.318) and in TMZ/PSi + PTT group (P = 0.003), whereas the cell viability was more significantly decreased in TMZ/PSi + PTT + HBO (P = 0.000063) group than that in other groups. Consistent with results in NCH-421K cells, PTT and HBO increased the sensitivity of TMZ/PSi against glioma stem-like C6 cells (Additional file 1: Figure S4).

In our study, although the amount of TMZ loaded into PSi NPs is the same with free TMZ in the treatment, nanoparticle-delivered TMZ exerted much higher cytotoxicity. Thermotherapy was thought to increase drug uptake into cells [22]. Hyperoxia can re-sensitize chemoresistant of human glioblastoma cells to TMZ [29]. Moreover, toxicity of combination treatment on morphology of NCH-421K was significant too. Tumorspheres of NCH-421K were fragmented significantly after treating with TMZ/PSi + PTT + HBO compared to other groups for 72 h (Fig. 2c, d). Therefore, PTT and HBO-adjuvanted with TMZ/PSi NPs enhance the effects on viability of glioma stem-like cells.

### Colony formation of NCH-421K cells after different treatments

**Fig. 3:**
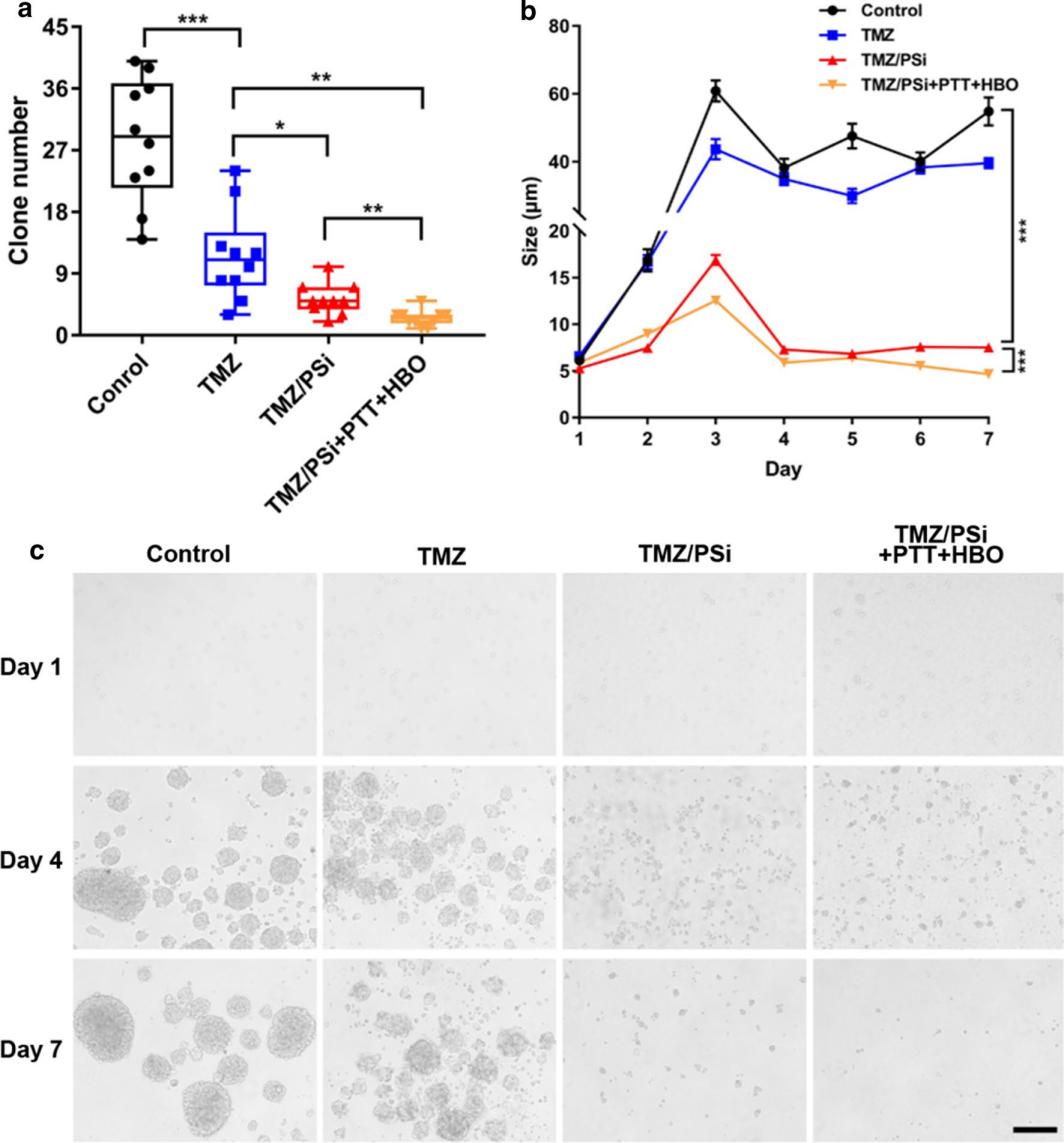
Colony formation of NCH-421K cells after different treatments. **a** Clone numbers after different treatments on day 7 (n = 10; *P < 0.05, **P < 0.01, ***P < 0.001). **b** Tumorsphere changes in 1 week after different treatments (n= 4; ***P < 0.001). **c** Images of tumorspheres after different treatments. NCH-421K cells at a density of 2.5 × 10^5^ cells/well were plated into 12-well plates and cultured overnight. Cells were treated with 100 μM TMZ or TMZ/PSi NPs for 24 h. Bar: 100 μm

Spheroid colony formation is a useful method to identify glioma stem cells (GSCs) [30]. In this study, we determined the effect of TMZ/PSi combined with PTT and HBO on GSC colony formation for 7 days (Fig. 3a, b, Additional file 1: Figure S5). Although TMZ alone attenuated GSC colony formation and tumorsphere growth, the remained GSCs were able to form tumorspheres. However, TMZ/PSi combined with PTT and HBO could inhibit GSCs self-renewal completely compared to TMZ alone (Fig. 3c). These studies demonstrate that TMZ/PSi combined with PTT and HBO can significantly suppress the clonogenic capacity of GSCs.

### mRNA and protein analysis in NCH-421K cells after different treatments

**Fig. 4:**
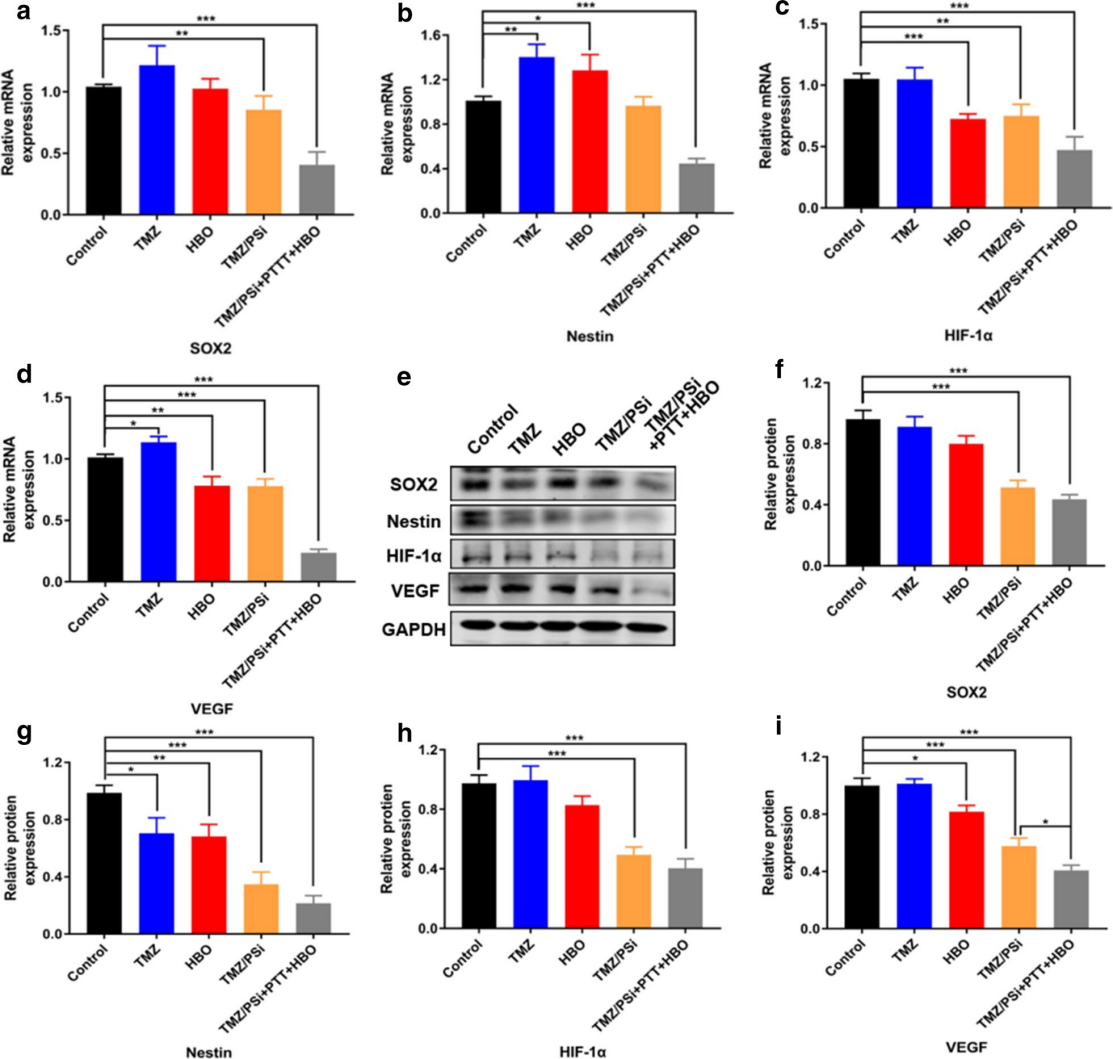
mRNA and protein analysis in NCH-421K cells after different treatments. **a**–**d** mRNA changes of SOX2, Nestin, HIF-1α and VEGF in NCH-421K cells after different treatments. NCH-421K cells were treated with 400 μM TMZ for 72 h (n = 3; *P < 0.05, **P < 0.01, ***P < 0.001). **e** Western blot analysis of SOX2, Nestin, HIF-1α and VEGF in NCH-421K cells after different treatments. NCH-421K cells were treated with 400 μM TMZ for 24 h. **f**–**i** The relative protein content of SOX2, Nestin, HIF-1α and VEGF in NCH-421K cells after different treatments (n = 3; *P < 0.05, **P < 0.01, ***P < 0.001)

To further investigate the underlying mechanism how TMZ/PSi combined with PTT and HBO eliminated GSCs, master regulators for the self-renewal and stemness maintenance of GSCs (SOX2 and Nestin) and hypoxia related molecules HIF-1α [31] and VEGF were evaluated. We investigated the expression of SOX2, Nestin, HIF-1α and VEGF through qRT-PCR and Western blot assays. As shown in Fig. 4, compared with the control group, TMZ increased the expression of stemness and hypoxia related factors (such as SOX2, Nestin, VEGF), which was caused closely related to chemo-resistance [32]. The expression of SOX2, HIF-1α, and VEGF were decreased in the TMZ/PSi group, which indicates nanomedicine TMZ/PSi can amend the drug resistance. HBO can improve the hypoxia in tumor microenvironment, thus the expression of HIF-1α and VEGF was decreased after treatment. All factor expressions were significant decreased in the combination treatment of PTT and HBO and TMZ/PSi, indicating that PTT and HBO can be as adjuvants for TMZ/PSi. The down-regulation of protein levels was not significant, whereas the decrease tendency of all factors expression was existed in the combination treatment compared to TMZ/PSi. The mRNA analysis in NCH-421K cells after treating with TMZ/PSi + HBO or TMZ/PSi + PTT were shown in Additional file 1: Figure S6. As shown in figure, no significant differences were observed in SOX2 (P > 0.05) and Nestin (P > 0.05) compared with TMZ/PSi. However, all 4 factor expressions were significantly decreased in the combination treatment of TMZ/PSi + HBO + PTT. This finding demonstrated that PTT and HBO alleviates the hypoxia in tumorspheres and increase the sensitivity of GSC to TMZ/PSi.

### Effects of TMZ/PSi combined with PTT and HBO on C6 cells

**Fig. 5:**
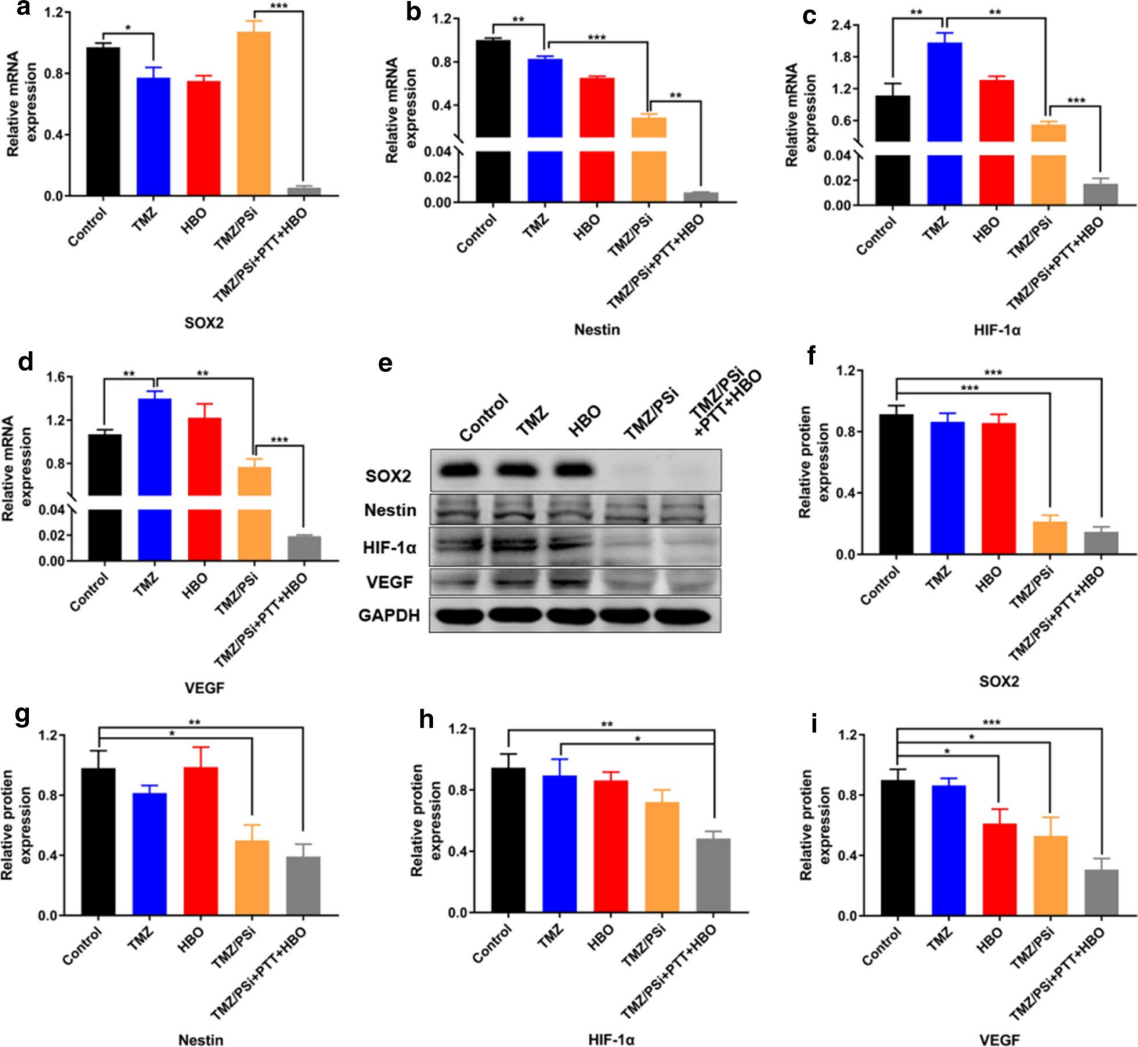
Effects of TMZ/PSi combined with PTT and HBO on C6 cells after different treatments. **a**–**d** mRNA changes of SOX2, Nestin, HIF-1α and VEGF in C6 cells after different treatments (n = 3; *P < 0.05, **P < 0.01, ***P < 0.001). C6 cells were treated with 400 μM TMZ for 72 h. **e** Western blot analysis of SOX2, Nestin, HIF-1α and VEGF in C6 cells after different treatments. C6 cells were treated with 400 μM TMZ for 24 h. **f**–**i** The relative protein content of SOX2, Nestin, HIF-1α and VEGF in C6 cells after different treatments (n = 3; *P < 0.05, **P < 0.01, ***P < 0.001)

To further investigate the effection of TMZ/PSi combined with PTT and HBO, C6 glioma cells were used. C6 glioma cells have properties in common with glioma stem cells [33, 34]. Most of the C6 cells are cancer stem-like cells with in vitro characteristics of self-renewal [35]. Therefore, C6 cells were chosen to further study the combination therapy effects of TMZ/PSi + PTT + HBO. As shown in Fig. 5, mRNA levels of SOX2, Nestin, HIF-1α and VEGF in C6 cells after combination treatment of TMZ/PSi + PTT + HBO were significantly decreased compare to the TMZ/PSi group (**P < 0.01; ***P < 0.001). Western blot results of SOX2, Nestin, HIF-1α and VEGF expression were decreased compare to the control group (**P < 0.01; ***P < 0.001). All factors showed significantly difference, which is similar to the results in NCH-421K after different treatments.

### Antitumor effects of TMZ/PSi NPs in combination with PTT and HBO

Glioma is a solid tumor characterized with hypoxia environment [26]. Tumor stem cells exhibit in hypoxic niches are known to be a key cause of the progression, metastasis and relapse [12]. Efficacy of hyperbaric oxygen therapy in combination with mild heat improved the anti-tumour effects of carboplatin [22]. In light of this, mild PTT and external nanodrug in treating glioma in vivo combined with HBO was performed. In this study, we choose C6 stem-like cells to establish tumor bearing mouse model to investigate the effects of combination treatment.

**Fig. 6:**
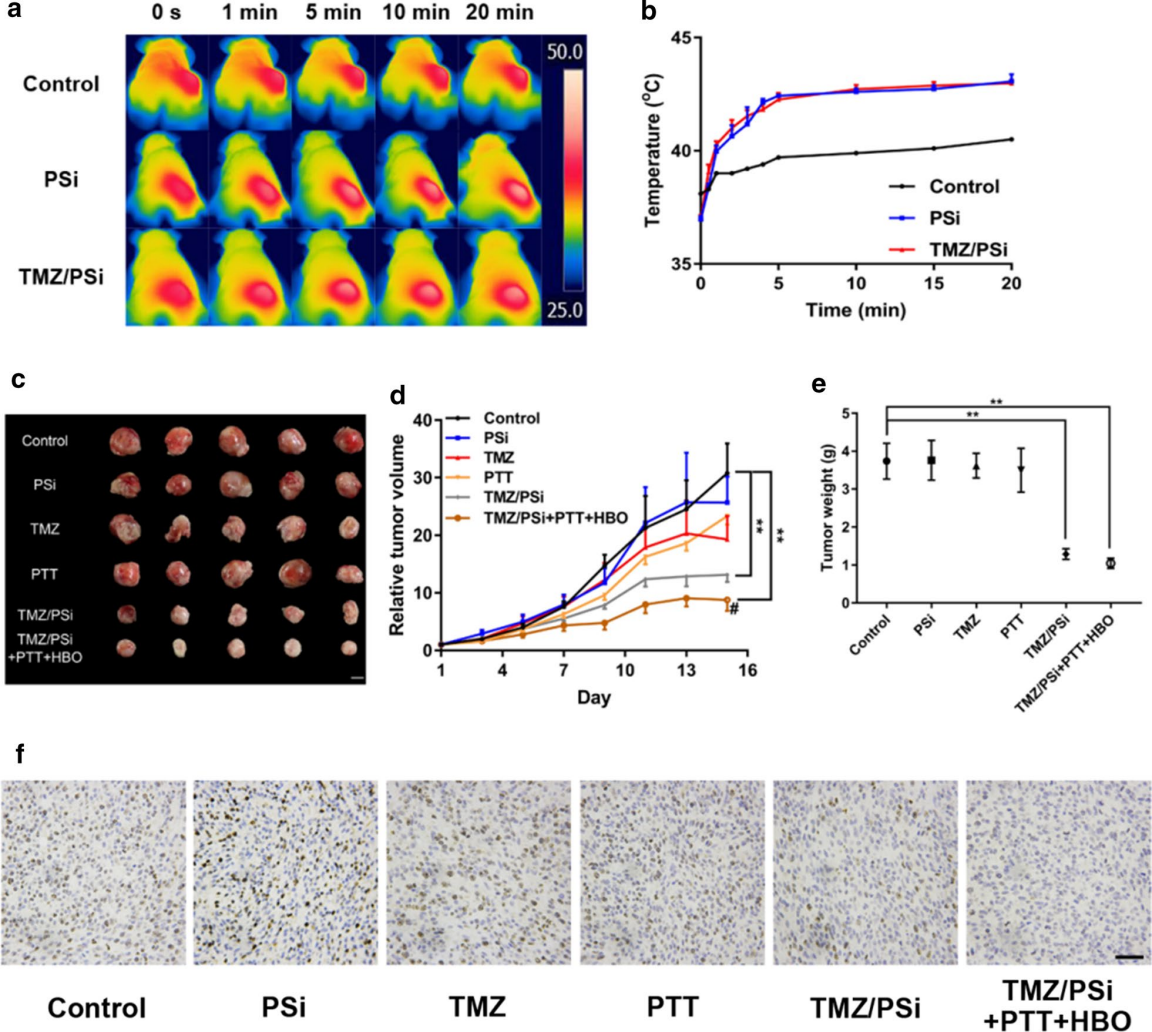
Antitumor effects of TMZ/PSi NPs in combination with PTT and HBO in vivo. **a** Tumor thermal imaging exposed to NIR irradiation. **b** Tumor thermal curve exposed to NIR irradiation. **c** Tumors from mice after different treatments. Bar: 1 cm. **d** Changes of tumor volume (n = 5; **P < 0.01, ^#^, TMZ/PSi vs TMZ/PSi + PTT + HBO, P < 0.05). **e** Tumor weight (n = 5; **P < 0.01). **f** Immunohistochemical staining of Ki-67, bar: 20 μm

The PSi NPs are well known to absorb IR energy and transfer it in the form of heat [36]. Upon NIR irradiation instantly after injection, the local tumor temperature gradually increased and reached about 43 °C (Fig. 6a, b), which was helpful to kill tumor cells. The mild hyperthermia regimes can avoid the massive necrosis that would be associated with high temperatures [28]. Therefore, PSi is an acceptable mild hyperthermia by patients. Figure 6d showed the relative tumor volume changes. There was no significant difference of tumor growth rate in PSi or PTT alone group, which suggested PSi or PTT had no obvious antitumor effect. Although free TMZ group had a tendency to inhibit the growth of tumors, there was no significant difference between the free TMZ group and control group, which indicated that free TMZ had little effect on tumor inhibition. However, the significant suppression of tumor growth was presented in TMZ/PSi group and TMZ/PSi combined with PTT and HBO group. Compared to TMZ/PSi group, better antitumor effects were shown in the TMZ/PSi combined with PTT and HBO group (^#^P < 0.05) (Fig. 6c). The tumor weight changes were in agreement with the tumor size changes (Fig. 6e). There were no significant changes of mouse body weights (Additional file 1: Figure S7). Based on the change of tumor volume, the tumor inhibition rate was calculated. The inhibition rates of PSi, TMZ and PTT were 16.5%, 37.2% and 24.2% respectively, whereas 57.2% in TMZ/PSi and 71.5% in TMZ/PSi + PTT + HBO were presented. Therefore, TMZ/PSi has a better anti-tumor effect than TMZ in vivo. PTT and HBO enhancing the effectiveness of TMZ/PSi, were useful as adjuvant therapy in the treatment of glioma. Our results disclosed that the combination treatment of TMZ/PSi NPs with PTT and HBO can efficiently augment the antitumor efficacy.

As shown in Fig. 6f, the proliferation of tumor cell (brown color) was relatively lower in the combination treatment than that in the other groups (Additional file 1: Figure S8). After H&E staining of tissues, no evident pathological changes were found (Additional file 1: Figure S9). Therefore, combination treatment of TMZ/PSi with PTT and HBO is a potential strategy for glioma therapy.

### Expression of tumor stem-like markers in vivo

**Fig. 7:**
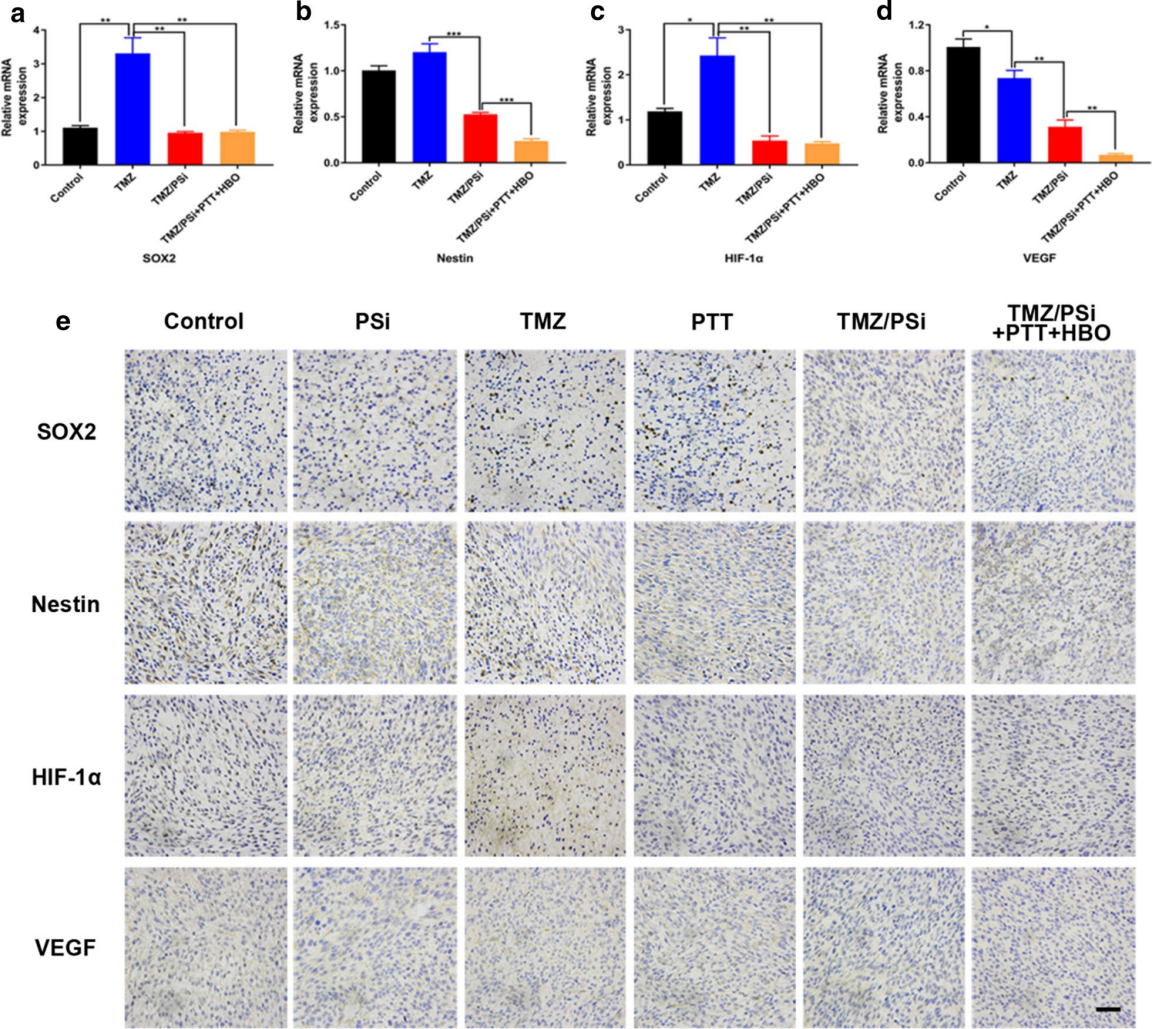
Expression of tumor stem-like markers in vivo. **a**–**d** mRNA changes of SOX2, Nestin, HIF-1α and VEGF in C6 tumor tissues after combination treatment (n = 3; *P < 0.05, **P < 0.01, ***P < 0.001). **e** Immunohistochemical staining for SOX2, Nestin, HIF-1α and VEGF of the tumors. Bar: 20 μm

Expression of tumor stem-like markers as well as other hypoxia related factors was determined in tumor tissues by the methods of immunohistochemistry and qRT-PCR. Studies have shown that low doses of TMZ can lead to chemoresistance in glioma cells [32, 37, 38]. As shown in Fig. 7a–c, compared with the control group, TMZ increased the expression of stemness and hypoxia related factors, which agrees with the reports. The expression of SOX2, Nestin, HIF-1α and VEGF in tumor tissues from TMZ/PSi combined with PTT and HBO group were efficiently decreased compared with other groups, suggesting PTT and HBO could effectively help to amend the tumor stemness and hypoxia environment (Fig. 7). The results of immunohistochemical staining were in line with the results of qRT-PCR (Fig. 7e), which is similar with the results in vitro. Therefore, the combination treatment is an effective strategy against glioma.

## Conclusion

How to reduce stemness and increase the drug sensitive is important for glioma therapy. In this study, HBO and mild thermotherapy can perfectly adjuvant the TMZ/PSi to amend the hypoxia environment in tumor and enhance TMZ/PSi therapy on stem-like cells in glioma. The combination treatment would be a potential treatment for glioma therapy.

## Additional file

**Additional file 1: Table S1.** List of primer sequences for mRNA analysis. **Figure S1.** The stability of TMZ/PSi in a week. **Figure S2.** The relative viability of NCH-421K cells after treatment with different concentration of TMZ (n = 5; **P < 0.01). **Figure S3.** The relative viability of C6 cells after treatment with different concentration of TMZ (n = 5; ***P < 0.001). **Figure S4.** The relative viability of C6 cells after treatment with PSi, TMZ, TMZ/PSi, or TMZ/PSi + PTT under normoxia environment or normoxia + HBO (n = 5; ***P < 0.001). **Figure S5.** The spheroid colony size of NCH-421K cells after different treatments on day 7 (n = 4; **P < 0.01, ***P < 0.001). **Figure S6.** mRNA analysis in NCH-421K cells after 72 h treatments (n = 3; *P < 0.05, **P < 0.01, ***P < 0.001). **Figure S7.** Relative body weight of the mice (n = 5). **Figure S8.** Relative optical density in each tumor slices of immunohistochemical staining (n = 5; **P < 0.01, ***P < 0.001). **Figure S9.** Histological sections of the mouse hearts, livers, spleens, lungs and kidneys. Bar: 20 μm.

## Abbreviations

PSi: porous silicon
TMZ: temozolomide
HBO: hyperbaric oxygen
CSC: cancer stem cell
ADMSC: adipose-derived mesenchymal stem cell
NIR: near-infrared
NPs: nanoparticles
PTT: photothermal therapy
TEM: transmission electron microscope
bFGF: basic fibroblast growth factor
EGF: epidermal growth factor
CCK-8: cell counting kit 8
qRT-PCR: quantitative real time polymerase chain reaction
GSCs: glioma stem cells
VEGF: vascular endothelial growth factor
HIF-1α: hypoxia inducible factor-1
H&E: hematoxylin & eosin staining
