# Supplementary material for: Mild thermotherapy and hyperbaric oxygen enhance sensitivity of TMZ/PSi nanoparticles via decreasing the stemness in glioma
Source: J Nanobiotechnology. 2019 Apr 1;17:47. doi: 10.1186/s12951-019-0483-1 (PMC6442425; doi:10.1186/s12951-019-0483-1)
Supplement: Supplementary file 1 — Additional file 1: Table S1. List of primer sequences for mRNA analysis. Figure S1. The stability of TMZ/PSi in a week. Figure S2. The relative viability of NCH-421K cells after treatment with different concentration of TMZ (n = 5; **P < 0.01). Figure S3. The relative viability of C6 cells after treatment with different concentration of TMZ (n = 5; ***P < 0.001). Figure S4. The relative viability of C6 cells after treatment with PSi, TMZ, TMZ/PSi, or TMZ/PSi + PTT under normoxia environment or normoxia + HBO (n = 5; ***P < 0.001). Figure S5. The spheroid colony size of NCH-421K cells after different treatments on day 7 (n = 4; **P < 0.01, ***P < 0.001). Figure S6. mRNA analysis in NCH-421K cells after 72 h treatments (n = 3; *P < 0.05, **P < 0.01, ***P < 0.001). Figure S7. Relative body weight of the mice (n = 5). Figure S8. Relative optical density in each tumor slices of immunohistochemical staining (n = 5; **P < 0.01, ***P < 0.001). Figure S9. Histological sections of the mouse hearts, livers, spleens, lungs and kidneys. Bar: 20 μm. [file 12951_2019_483_MOESM1_ESM.docx]

Supplementary Material

Supplementary figures and table.

**Table S1 Primer sequences**

| Primer | Sequence |
| --- | --- |
| GAPDH-F（human NCH-421K cell） | GGAGCGAGATCCCTCCAAAAT |
| GAPDH-R（human NCH-421K cell） | GGCTGTTGTCATACTTCTCATGG |
| SOX2-F（human NCH-421K cell） | TACAGCATGTCCTACTCGCAG |
| SOX2-R（human NCH-421K cell） | GAGGAAGAGGTAACCACAGGG |
| Nestin-F（human NCH-421K cell） | GAAGGGCAATCACAACAGGTG |
| Nestin-R（human NCH-421K cell） | GGGGCCACATCATCTTCCA |
| HIF-1α-F（human NCH-421K cell） | GAACGTCGAAAAGAAAAGTCTCG |
| HIF-1α-R（human NCH-421K cell） | CCTTATCAAGATGCGAACTCACA |
| VEGF-F（human NCH-421K cell） | AGGGCAGAATCATCACGAAGT |
| VEGF-R（human NCH-421K cell） | AGGGTCTCGATTGGATGGCA |
| GAPDH-F (rat C6 cell) | GTTCCTACCCCCAATGTGTCC |
| GAPDH-R (rat C6 cell) | TAGCCCAAGATGCCCTTCAGT |
| Nestin-F (rat C6 cell) | AGAGTCAGATCGCTCAGATCC |
| Nestin-R (rat C6 cell) | GCAGAGTCCTGTATGTAGCCAC |
| SOX2-F (rat C6 cell) | CGGCACAGATGCAACCGAT |
| SOX2-R (rat C6 cell) | CCGTTCATGTAGGTCTGCG |
| HIF-1α-F (rat C6 cell) | GTCCCAGCTACGAAGTTACAGC |
| HIF-1α-R (rat C6 cell) | CAGTGCAGGATACACAAGGTTT |
| VEGF-F (rat C6 cell) | GCACATAGAGAGAATGAGCTTCC |
| VEGF-R (rat C6 cell) | CTCCGCTCTGAACAAGGCT |


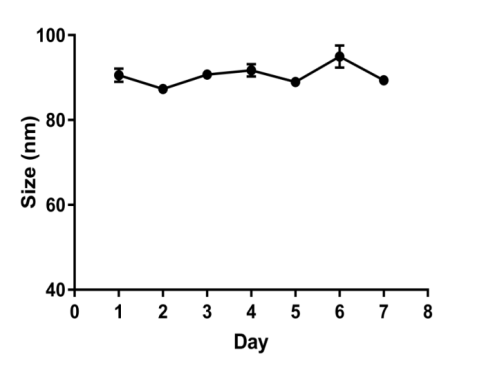


Figure S1. The stability of TMZ/PSi in a week.


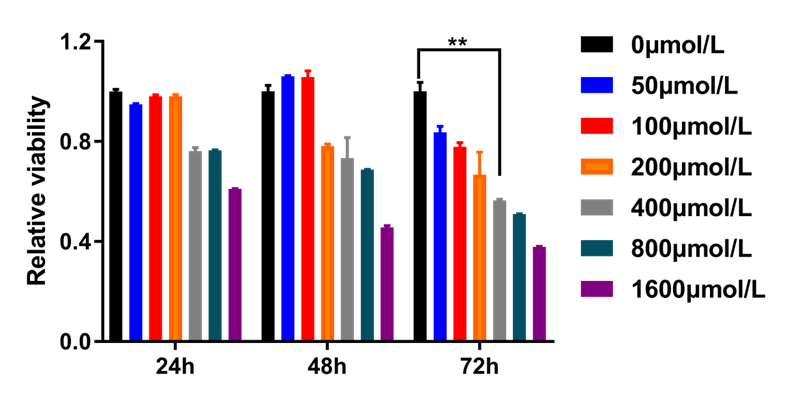


Figure S2. The relative viability of NCH-421K cells after treatment with different concentration of TMZ (n= 5; **P < 0.01).


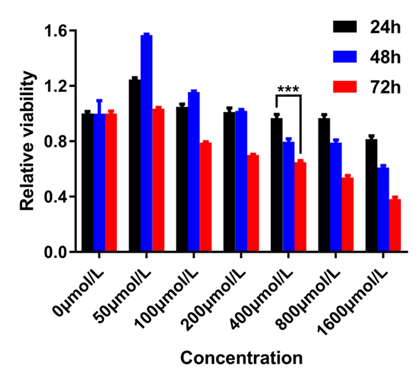


Figure S3. The relative viability of C6 cells after treatment with different concentration of TMZ (n= 5; ***P < 0.001).


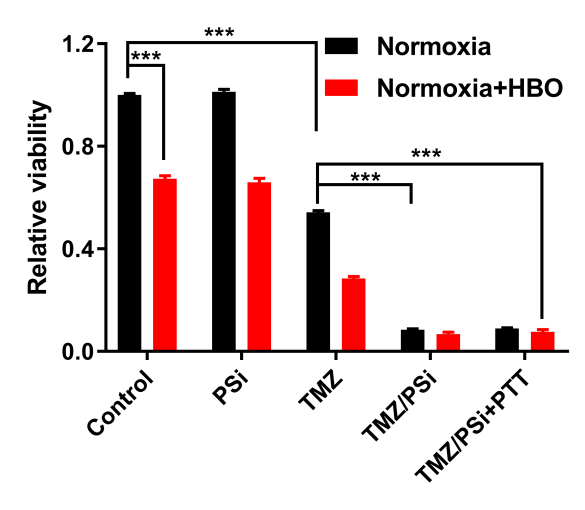


Figure S4. The relative viability of C6 cells after treatment with PSi, TMZ, TMZ/PSi, or TMZ/PSi+PTT under normoxia environment or normoxia+HBO (n= 5; ***P < 0.001).


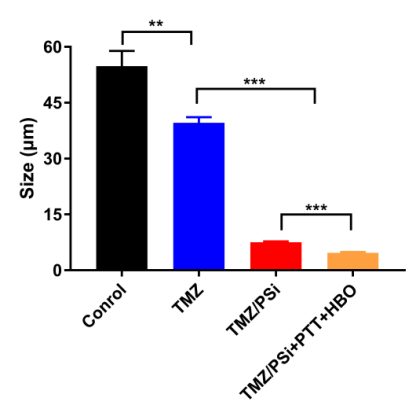


Figure S5.The spheroid colony size of NCH-421K cells after different treatments on day 7 (n= 4; **P < 0.01, ***P < 0.001).


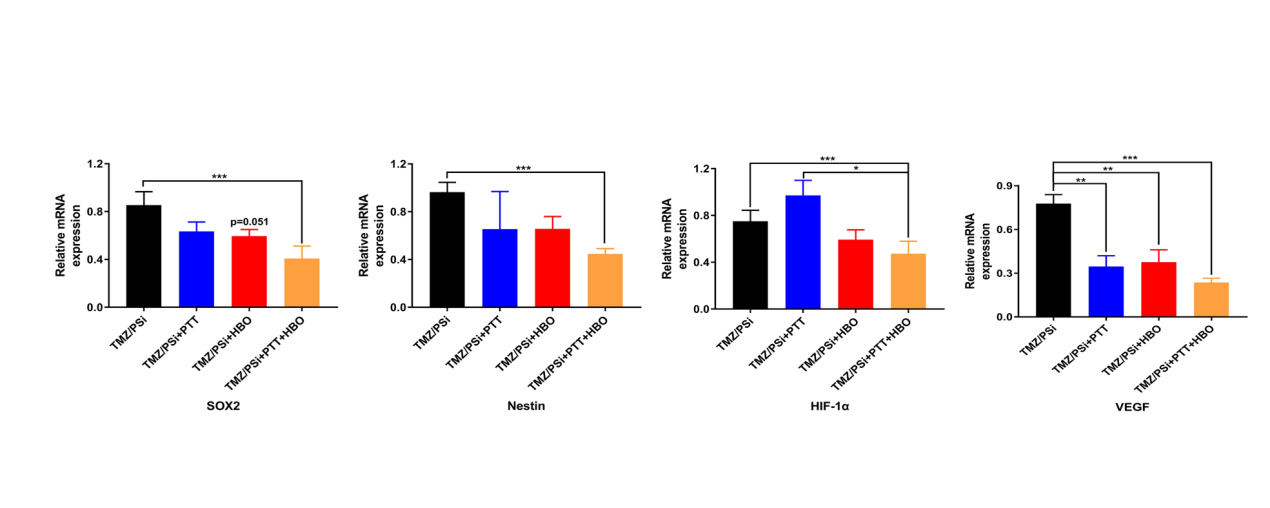


Figure S6. mRNA analysis in NCH-421K cells after 72h treatments (n= 3; *P < 0.05, **P < 0.01, ***P < 0.001).


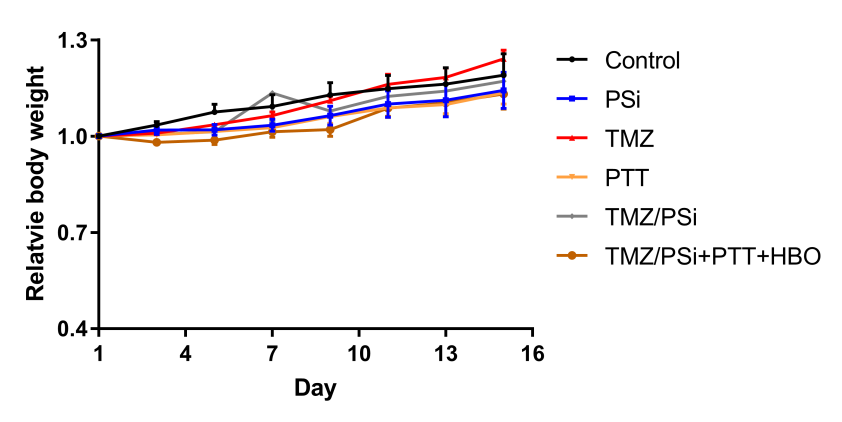


Figure S7. Relative body weight of the mice (n= 5).


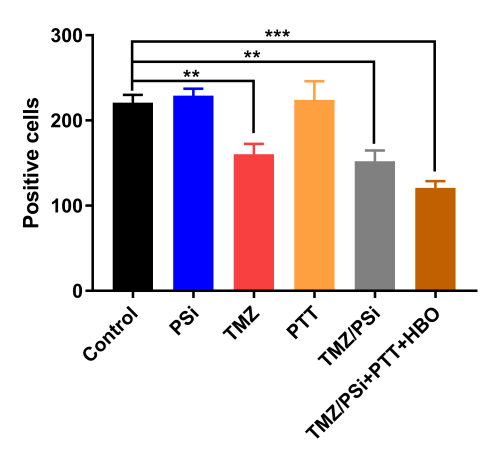


Figure S8. Relative optical density in each tumor slices of immunohistochemical staining (n= 5; **P < 0.01, ***P < 0.001).


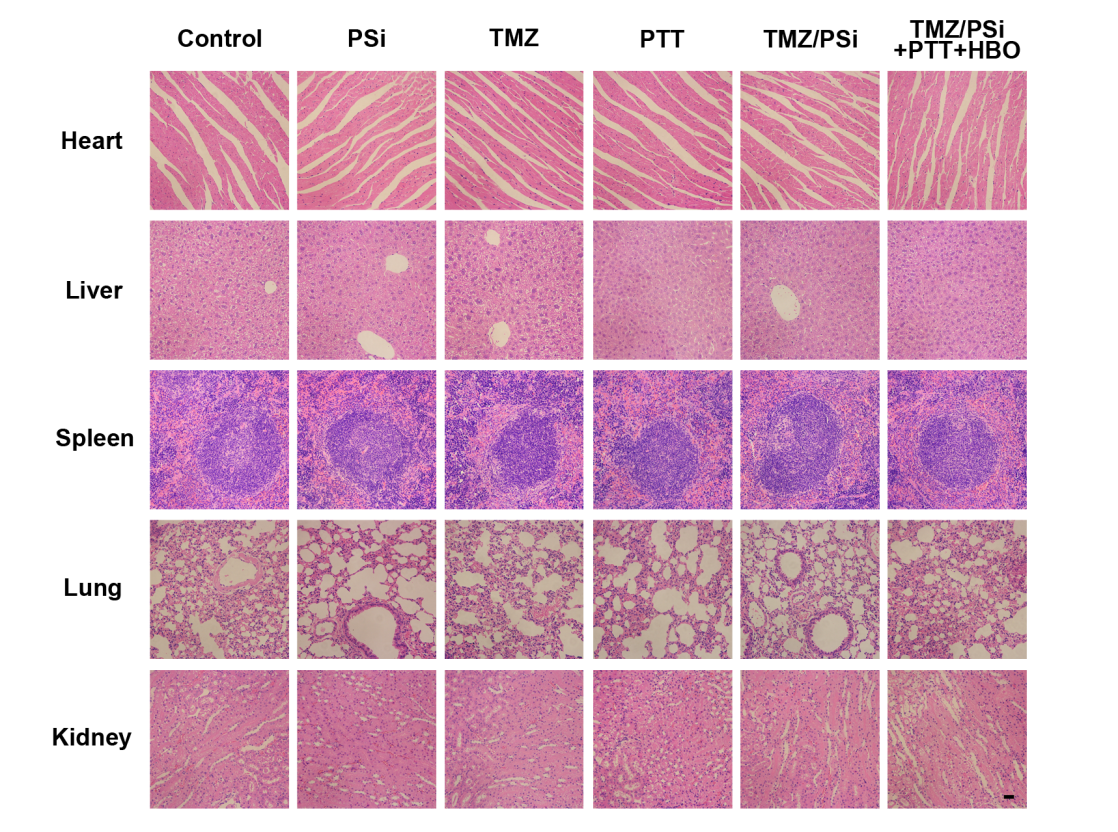


Figure S9. Histological sections of the mouse hearts, livers, spleens, lungs and kidneys. Bar：20μm.
